# Dissecting the Gene Dose-Effects of the *APOE* ε4 and ε2 Alleles on Hippocampal Volumes in Aging and Alzheimer’s Disease

**DOI:** 10.1371/journal.pone.0054483

**Published:** 2013-02-06

**Authors:** Christopher A. Hostage, Kingshuk Roy Choudhury, Pudugramam Murali Doraiswamy, Jeffrey R. Petrella, for the Alzheimer’s Disease Neuroimaging Initiative

**Affiliations:** 1 Department of Radiology, Duke University School of Medicine, Durham, North Carolina, United States of America; 2 Department of Psychiatry and the Duke Institute for Brain Sciences, Duke University School of Medicine, Durham, North Carolina, United States of America; McGill University/Douglas Mental Health Univ. Institute, Canada

## Abstract

**Objective:**

To investigate whether there is a specific dose-dependent effect of the Apolipoprotein E (*APOE*) ε4 and ε2 alleles on hippocampal volume, across the cognitive spectrum, from normal aging to Alzheimer’s Disease (AD).

**Materials and Methods:**

We analyzed MR and genetic data on 662 patients from the Alzheimer’s Disease Neuroimaging Initiative (ADNI) database–198 cognitively normal controls (CN), 321 mild-cognitive impairment (MCI) subjects, and 143 AD subjects–looking for dose-dependent effects of the ε4 and ε2 alleles on hippocampal volumes. Volumes were measured using a fully-automated algorithm applied to high resolution T1-weighted MR images. Statistical analysis consisted of a multivariate regression with repeated-measures model.

**Results:**

There was a dose-dependent effect of the ε4 allele on hippocampal volume in AD (*p* = 0.04) and MCI (*p* = 0.02)–in both cases, each allele accounted for loss of >150 mm^3^ (approximately 4%) of hippocampal volume below the mean volume for AD and MCI subjects with no such alleles (Cohen’s *d* = −0.16 and −0.19 for AD and MCI, respectively). There was also a dose-dependent, main effect of the ε2 allele (*p*<0.0001), suggestive of a moderate protective effect on hippocampal volume–an approximately 20% per allele volume increase as compared to CN with no ε2 alleles (Cohen’s *d* = 0.23).

**Conclusion:**

Though no effect of ε4 was seen in CN subjects, our findings confirm and extend prior data on the opposing effects of the *APOE* ε4 and ε2 alleles on hippocampal morphology across the spectrum of cognitive aging.

## Introduction

Apolipoprotein E (ApoE), aside from its well-known role as a lipid-transporting entity, plays a key role in many CNS-related processes [1]. Of the three alleles for the *APOE* gene (ε2, ε3, and ε4), inheritance of the epsilon 4 (ε4) variant is well-established as the most important genetic risk factor for the development of late-onset Alzheimer’s Disease (AD): presence of ε4 is associated with a higher risk for the development of AD, earlier age-of-onset, and interacts with gender, age, and race to accelerate progression [2]. Conversely, the ε2 variant has been repeatedly shown to have nearly perfectly-opposing effects: it is markedly underrepresented in AD cases, and is associated with a delayed age-of-onset of AD as well as with decreased amounts of AD-related brain pathology in affected patients [3]–[5].

Consequently, there has been much concentration recently on discerning the mechanisms by which ApoE might act to influence AD pathophysiology. For instance, many lines of evidence and argument make the case for a “negative gain-of-function” role of the ε4 isoform–including its association with impaired cognition, its inhibition of neurite sprouting processes, its tendency to predispose neuronal tissue to the deposition of *tau* tangles, and its possible direct neurotoxic effects [6]. It also appears to be less able than the ε2 and ε3 isoforms to fulfill its role in the breakdown of extracellular amyloid-β peptide in the brain [7], [8]. On the other hand, it has also been argued that ApoE in general serves primarily neurotrophic and neuro-protective roles, and that associations of ε4 with higher risk for and more disease is due more to absence of the ε2/3 isoforms than it is the presence of ε4–a more than semantic distinction [9].

A large body of work has thus been focused on probing the various facets of ApoE’s relationship to AD pathology. One such facet that still remains uncertain is whether there is an effect of *APOE* genotype on structural brain changes seen in AD, such as hippocampal atrophy. Many prior studies have examined the hippocampi in particular with regard to volumes and/or atrophy rates as affected by the presence of ε4; however, one can find both a large set of studies which demonstrate an effect [10]–[14], as well as a similarly large set which does not [15]–[20]. There are several limitations in most of the above-mentioned studies, including low sample sizes and reliance on manual or semi-automated methods of volume-derivation. Furthermore, even less is known about any potential dose-dependent effect of the *APOE* ε4 allele on hippocampal volumes; a dose-effect, if found, would add to our understanding of the mechanisms whereby the ε4 isoform of ApoE accelerates the pathophysiology of AD. Further, structural MR effects of the ε2 isoform have not been extensively studied, and those studies that have examined this issue–including a recent study by Chiang GC *et al* [21]–have been unable to demonstrate any effect on hippocampal size [12], [13], [22].

For the current study we examined a large cohort (*n* = 662) of subjects drawn from a publicly available database, made up of cognitively normal (CN) controls, subjects with mild-cognitive impairment (MCI), and subjects with AD, in order to investigate the differential effects of the *APOE* ε4 and ε2 alleles on hippocampal volume across the cognitive spectrum–and in particular whether any such effects are dose-dependent.

## Materials and Methods

### Ethics Statement

The ADNI study was approved by IRBs of all participating sites including the Duke University Health System IRB. All subjects and if applicable, their legal representatives, gave written informed consent prior to the collection of clinical, genetic and imaging data. The Duke University Health System (DUHS) Institutional Review Board determined that the current analysis of the ADNI database met the definition of research not involving human subjects as described in 45 CFR 46.102(f), 21 CFR 56.102(e) and 21 CFR 812.3(p) and satisfies the Privacy Rule as described in 45CFR164.514 (DUHS Protocol ID: Pro00034962).

### Subjects

The data used in this study were obtained from a database compiled for the Alzheimer’s Disease Neuroimaging Initiative (ADNI) (adni.loni.ucla.edu) [23], a large multi-center natural history trial whose goal has been to examine the ability of MRI, PET, biologic markers, and clinical/neuropsychological assessments to be used in combination to measure progression in MCI and AD. Please see www.adni-info.org for up-to-date information.

Subjects whose data were selected for analysis in the current study were required to have information for all of the following available in the ADNI database: age, race, gender, and years of education; an MMSE score obtained at the baseline visit; *APOE* genotyping results; baseline-visit 1.5 T MRI scans which were analyzed for ADNI by Freesurfer software, version 4.4; and a measure of estimated intracranial volume (ICV) derived from their baseline MR scan. As of 11/30/2011, 662 subjects from the ADNI-1 arm met criteria and were thus included: 198 CN controls, 321 MCI subjects, and 143 AD subjects. Due to the putative opposing effects of the ε4 and ε2 alleles, individuals with the *APOE* ε4/ε2 or ε2/ε4 genotype (*n = *14) were excluded. For a breakdown by *APOE* allele load see Tables 1–2.

**Table 1:** Breakdown of subjects by diagnosis and *APOE* epsilon 4 status.

	ε4 = 0	ε4 = 1	ε4 = 2	total
**CN**	143	50	5	*198*
**MCI**	153	127	41	*321*
**AD**	50	62	31	*143*
Total	*346*	*239*	*77*	***662***

CN = cognitively normal, MCI = mild cognitive Impairment, AD = Alzheimer’s Disease, ε4 = APOE epsilon 4.

**Table 2:** Breakdown of subjects by diagnosis and *APOE* epsilon 2 status.

	ε2 = 0	ε2 = 1	ε2 = 2	total
**CN**	170	26	2	*198*
**MCI**	307	14	0	*321*
**AD**	139	4	0	*143*
total	*616*	*44*	*2*	***662***

CN = cognitively normal, MCI = mild cognitive Impairment, AD = Alzheimer’s Disease, ε2 = APOE epsilon 2.

### Classification, Clinical Diagnosis, and APOE Genotyping

Subjects who met eligibility criteria for the ADNI database were assessed with a standardized protocol in order to measure cognition and assign subjects to diagnostic category [24]. For the ADNI, to be classified as CN, the subject had an MMSE score between 24–30 (inclusive), a clinical dementia rating scale (CDR) of 0, was required to meet specific cutoffs for Wechsler Memory Scale Logical Memory II-Delayed Paragraph Recall score (specifically: >or = to 9 for subjects with 16 or more years of education; >or = to 5 for subjects with 8–15 years of education; and >or = to 3 for 0–7 years of education), and had to be non-depressed, non-MCI, and non-demented. In order to be classified in the MCI group, the subject needed an MMSE score between 24–30 (inclusive), a memory complaint, objective evidence of memory loss as measured by their Wechsler Memory Scale Logical Memory II-Delayed Paragraph Recall score (specifically: <9 for subjects with 16 or more years of education; <5 for subjects with 8–15 years of education; and <3 for subjects with 0–7 years of education ), a CDR of 0.5, absence of significant levels of impairment in cognitive domains other than memory, preserved activities of daily living, and absence of dementia. Finally, a subject was classified in the AD group if he or she had an MMSE score between 20–26 (inclusive), a CDR of 0.5 or 1.0, and also met National Institute of Neurological and Communicative Disorders and Stroke and Alzheimer’s Disease and Related Disorders Association (NINCDS/ADRDA) criteria for probable AD [25]. Specific exclusion criteria for ADNI-1 were: presence of other significant neurologic disease; baseline MR scans showing evidence of infection, infarction, or other focal lesion or multiple lacunes; those with pacemakers, aneurysm clips or other devices which prevent them from receiving MRI scans; presence of major depression, psychotic features, alcohol/substance abuse or dependence in previous two years; significant medical illness or laboratory abnormalities (B12, RPR, TFTs) that might have interfered with the study; residence in a skilled nursing facility; or current use of warfarin or certain other psychoactive medications. More information can be found by referring to the ADNI-1 Procedures manual [26].

Genotyping of all subjects for *APOE* allele status was performed using DNA extracted from peripheral blood cells. The cells were collected in 1 EDTA plastic tubes (10 mL) and sent by express mail to the University of Pennsylvania AD Biofluid Bank Laboratory by overnight delivery at room temperature. Please see the ADNI-1 Procedures manual for more detailed information [26].

### MR Imaging Acquisition

The ADNI used 1.5T MP-RAGE MR images that were then pre-processed, undergoing correction for gradient non-linearity via “GradWarp”, intensity non-uniformity using B1 calibration scans, and residual intensity non-uniformity using “N3.” The images also underwent scaling based on scans of a phantom device. All scans underwent rigorous vetting for quality control purposes, and were performed using a standardized protocol specifically developed for the ADNI, tailored for use with each model of scanner used at the different data collection sites [27]. The current study’s hippocampal volume analyses incorporated only information derived from each subject’s baseline, initial-visit MR scan using Freesurfer volumetry. More detailed information for the specific MR acquisition protocols for each type of scanner used can be found at the ADNI@LONI website [28].

### MR Volume Derivations

**Figure 1:**
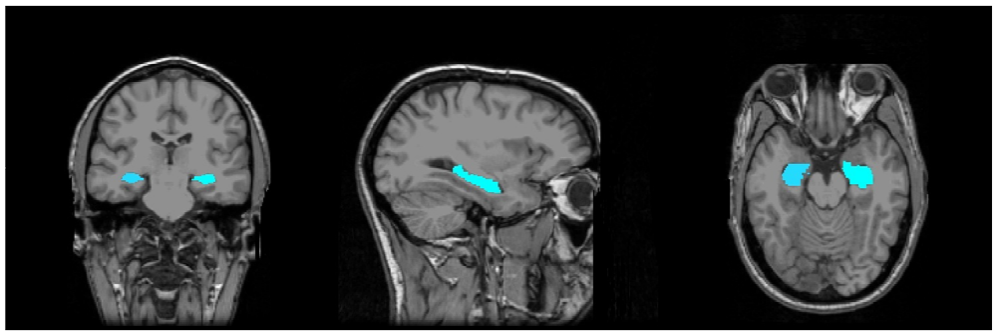
Freesurfer hippocampal region of interest. Coronal, sagittal, and axial (1.5T) T1-weighted MR images from the Freesurfer Image Analysis Suite of sample subject “Bert”, with color overlays depicting the extent of the Freesurfer hippocampal region of interest used to derive hippocampal volumes.

Volumetric segmentation was performed with the Freesurfer image analysis suite (Figure 1), which has been previously validated as an automated method through which to derive sub-cortical volumes. Freesurfer volumetry uses a combination of intensity mapping and probabilistic spatial atlases [29] in order to use multiple sources of information in assigning tissue labels on a voxel-by-voxel basis to MR scans; this method has been shown to produce volumetric determinations statistically indistinguishable from those provided by manual segmentation, and has also been shown to be able to reliably detect the subtle structural changes that are present early in Alzheimer’s Disease [30]. The Freesurfer segmentation process includes motion correction of volumetric T1-weighted images, removal of non-brain tissue using a hybrid watershed/surface deformation procedure [31], automated Talairach transformation, segmentation of the subcortical white matter and deep gray matter volumetric structures (including hippocampus, amygdala, caudate, putamen, ventricles) [30] intensity normalization [32], tessellation of the gray matter white matter boundary, automated topology correction [33]–[34] and surface deformation following intensity gradients to optimally place the gray/white and gray/cerebrospinal fluid borders at the location where the greatest shift in intensity defines the transition to the other tissue class [35]–[37]. The specific details of the automatic parcellation of subcortical gray matter structures such as the hippocampus using Freesurfer have been described previously in [30]. Freesurfer morphometric procedures have been demonstrated to show good test-retest reliability across scanner manufacturers and across field strengths [38].

The derivation of subject total intra-cranial volume (ICV) was done at the University of California-San Diego, and made available in the ADNI database [39]–[40]. Briefly, “estimated intracranial volume” (ICV) is calculated based on a mask. To generate the mask, the baseline image is automatically segmented; all thus-defined brain and ventricular voxels are given the value “1,” and all other voxels a value of “0″. This binary mask is then repeatedly smoothed with a Gaussian kernel to produce a simply-connected uniform mask, covering all sulci, whose boundary tapers smoothly from 1 to 0 over the length of a few voxels. Ideally, the mask would end on the skull and include the brain stem to the point where it begins to bend within the neck. The smoothing can be controlled to begin tapering at the skull, so that voxels with a mask value less than 1 can be considered outside the ICV and therefore ignored.

### Statistical Analyses

Statistical analyses were performed using the R Statistical package (www.R-project.org). All comparisons and test statistics were assessed for significance using α = 0.05 given our a priori hypotheses.

For cohort characterization, baseline mean values for age, years of education, and MMSE score according to both *APOE* ε4 and *APOE* ε2 dose were calculated, along with the distributions by gender and race. Overall *APOE* ε4 and ε2 allele frequencies were calculated, and differences in allelic frequency among the diagnostic groups was examined using Chi-square analysis or Fisher’s exact test, as appropriate. Differences in baseline cognitive functioning between the diagnostic categories as measured by Wechsler Memory Scale Logical Memory II-Delayed Paragraph Recall mean scores were explored using ANOVA, with Tukey-Kramer HSD *post hoc* analysis.

Preliminary analysis of hippocampal volumes was done in order to describe the baseline distribution of unadjusted mean hippocampal volumes by *APOE* status within each diagnostic category.

In order to determine if significant differences in hippocampal volume existed between *APOE* cohorts, we modeled hippocampal volume utilizing a linear repeated-measures model (which assumes that left and right hippocampal volumes on the same individual are correlated), i.e. multivariate regression involving the following predictors: age, intra-cranial volume (ICV), diagnosis, left- or right sided measurement, *APOE* genotype, and their cross-products; only significant interaction terms were retained. Gender and race effects were not included in this analysis. Our analysis treated *APOE* genotype as a continuous numeric variable to examine any dose-dependency (Model 1.1). For clarity, we will define “dose-dependent” to mean demonstration that a given allele has a statistically significant effect when it is considered as a continuous variable, i.e. being homozygous confers twice as much effect on hippocampal volume as being heterozygous.
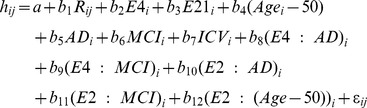
(Model 1.1)


Model 1.1 thus fits the available data under the assumption of dose-dependency of *APOE* allele copy number. In Model 1.1, h_ij_ represents the hippocampal volume for the i-th subject, i = 1,…,662 and the j-th side, j = 1 if left and = 2 if right. R_ij_ = 0 if j = 1, = 1 if j = 2, indicates the side measured. E4_i_ represents the number of alleles (0, 1 or 2) of the ApoE4 gene for the i-th subject. E2_i_ represents the number of alleles (0, 1, or 2) of the ApoE2 gene for the i-th subject. Age_i_ is the age of the i-th subject in years. AD_i_ = 1 if the i-th subject has a diagnosis of Alzheimer’s, = 0 otherwise. MCI_i_ = 1 if the i-th subject has a diagnosis of mild cognitive impairment, = 0 otherwise. ICV_i_ is the total cranial volume of the i-th subject, standardized by subtracting the mean and then dividing by the standard deviation across all subjects. The operator “:” denotes an interaction between respective terms. The error term ε_ij_ is assumed have a Gaussian distribution and to be uncorrelated across subjects i. It is also assumed to have a fixed correlation coefficient ε for measurements the two sides of the brain on the same subject.

## Results

### Demographic, Cognitive, and Genetic Characteristics and Preliminary Hippocampal Volume Analysis

Results for the analysis of baseline MMSE and demographic characteristics according to *APOE* ε4 and ε2 dose are summarized in Tables 3 and 4. The mean scores for the Wechsler Memory Scale Logical Memory II subscale (Delayed Recall) were, for CN, MCI, and AD subjects respectively (mean ±1 standard error of the mean): 12.90±0.19, 3.82±0.15, and 1.48±0.23; ANOVA with Tukey-Kramer HSD *post-hoc* analysis revealed that each of these means was statistically significantly different from both others (*p*<0.001 for all three diagnosis-wise comparisons).

**Table 3:** Demographic and cognitive summary by ε4.

	Parameter	ε 4 = 0	ε4 = 1	ε4 = 2
CN	n =	*143*	*50*	*5*
n = 198	Age	76.2±0.4	75.7±0.7	74.6±2.3
	Years of Ed.	16.1±0.2	16.1±0.4	16.2±1.2
	MMSE	29.1±0.1	29.3±0.1	29.0±0.4
	Gender (m/f)	78/65	26/24	3/2
	Race (Asian/Black/White)	1/9/133	0/3/47	1/0/4
MCI	n =	*153*	*127*	*41*
n = 321	**Age**	**75.9±0.6**	**74.6±0.6**	**71.7±1.1**
	Years of Ed.	15.8±0.2	15.6±0.3	15.7±0.5
	MMSE	27.2±0.1	27.0±0.2	26.7±0.3
	Gender (m/f)	98/55	81/46	24/17
	Race (Asian/Black/White)	6/4/143	3/6/118	0/1/40
AD	n =	*50*	*62*	*31*
n = 143	**Age**	**76.3±1.0**	**75.9±0.9**	**71.7±1.3**
	Years of Ed.	15.4±0.4	14.5±0.4	14.4±0.6
	MMSE	23.4±0.3	23.5±0.2	23.5±0.4
	Gender (m/f)	24/26	33/29	18/13
	Race (Asian/Black/White)	2/1/47	0/3/59	0/1/30

All means are reported ± 1 standard error. All **bolded** parameters were significant according to ANOVA, Chi-square analysis, or Fisher’s Exact Test as appropriate. CN = cognitively normal, MCI = mild cognitive Impairment, AD = Alzheimer’s Disease, ε4 = APOE epsilon 4.

**Table 4:** Demographics and Cognitive Summary by ε2.

	Parameter	ε2 = 0	ε2 = 1	ε2 = 2
CN	n* = *	*170*	*26*	*2*
n = 198	Age	76.2±0.4	75.2±1.0	73.5±3.6
	Years of Ed.	16.2±0.2	15.5±0.5	18.0±1.9
	MMSE	29.2±0.1	28.8±0.2	30.0±0.7
	Gender (m/f)	93/77	12/14	2/0
	**Race (Asian/Black/White)**	**2/7/161**	**0/4/22**	**0/1/1**
MCI	n* = *	*307*	*14*	*0*
n = 321	Age	74.8±0.4	76.9±1.9	–
	Years of Ed.	15.7±0.2	15.9±0.8	–
	MMSE	27.0±0.1	27.4±0.5	–
	Gender (m/f)	197/110	6/8	–
	Race (Asian/Black/White)	8/11/288	1/0/13	–
AD	n* = *	*139*	*4*	*0*
n = 143	Age	75.1±0.6	75.5±3.8	–
	Years of Ed.	14.8±0.3	14.5±1.6	–
	MMSE	23.5±0.2	22.7±0.7	–
	Gender (m/f)	74/65	1/3	–
	Race (Asian/Black/White)	2/5/132	0/0/4	–

All means are reported ± 1 standard error. All **bolded** parameters were significant according to ANOVA, Chi-square analysis, or Fisher’s Exact Test as appropriate. CN = cognitively normal, MCI = mild cognitive Impairment, AD = Alzheimer’s Disease, ε2 = APOE epsilon 2.

The overall *APOE* ε4 allele frequency was 0.297. The allele was underrepresented in CN subjects (*f* = 0.15) and overrepresented in AD subjects (*f* = 0.43)–this difference was significant by Chi-square analysis (*p*<0.001). The overall *APOE* ε2 frequency was 0.036. The allele was overrepresented in CN subjects (*f* = 0.076) and underrepresented in MCI and AD subjects (*f* = 0.022 and 0.014, respectively)–this difference was significant by Fisher’s exact test (*p* = 0.001).

Results for preliminary analysis to describe baseline unadjusted mean hippocampal volumes for subjects included in the current study are summarized in Tables 5 and 6.

**Table 5:** Summary of unadjusted hippocampal volumes by *APOE* ε4 allele dose and diagnostic category.

Diagnosis	ε4 dose	Mean	SD	*n*	SE
CN	0	3341.5	442.0	286	26.1
CN	1	3282.9	411.2	100	41.1
CN	2	3477.1	278.1	10	88.0
MCI	0	2989.1	553.9	306	31.7
MCI	1	2846.8	521.3	254	32.7
MCI	2	2731.4	392.6	82	43.4
AD	0	2666.0	665.1	100	66.5
AD	1	2572.9	445.0	124	40.0
AD	2	2501.5	407.9	62	51.8

Unadjusted values. “ε4 dose” = copy number of APOE ε4 allele,

“SD” = standard deviation, “SE” = standard error of the mean. “Mean” units are mm^3^.

**Table 6:** Summary of unadjusted hippocampal volumes by *APOE* ε2 allele dose and diagnostic category.

Diagnosis	ε2 dose	Mean	SD	*n*	SE
CN	0	3317.2	417.5	340	22.6
CN	1	3342.4	396.2	52	54.9
CN	2	4269.3	983.3	4	491.6
MCI	0	2895.0	529.0	614	21.3
MCI	1	3006.5	557.7	28	105.4
MCI	2			0	
AD	0	2599.8	517.2	278	31.0
AD	1	2249.8	788.4	8	278.7
AD	2			0	

Unadjusted values. “ε2 dose” = copy number of APOE ε2 allele,

“SD” = standard deviation, “SE” = standard error of the mean. “Mean” units are mm^3^.

### Hippocampal Volume Model

In the hippocampal volume model (Model 1.1), we first confirmed the assumption of Gaussianity using a *q*-*q* plot of the residuals–this appeared to hold quite well. In Model 1.1, age, diagnosis, ICV, and side (Right or Left Hippocampus) were significant predictors (*p*<0.0001 for all). The mean age-effect was a loss of 23.57 mm^3^ of hippocampal volume per year. Right-sided volumes were larger on average by 41.26 mm^3^. All other factors equal, individuals with MCI had hippocampal volumes that were smaller by 358.29 mm^33^ as compared to CN controls (−10.8%); for AD subjects, this figure was 569.5 mm^3^ (−17.2%). There were no other significant effects, aside from the *APOE*-driven effects which are discussed below. See Table 7 for a full summary of the results of Hippocampal Volume Model 1.1.

**Table 7:** Summary of effects for variables on hippocampal volume: *APOE* dose-dependent model.

	Effect (mm^3^)	*p*-value
**(Intercept)**	3893.44	<0.0001
**Right side**	41.27	<0.0001
***ApoE4***	−2.97	0.96
***ApoE2***	769.33	<0.0001
**Age**	−23.31	<0.0001
**AD**	−560.75	<0.0001
**MCI**	−349.72	<0.0001
**ICV**	184.78	<0.0001
***ApoE4*** **:AD**	−152.66	0.04
***ApoE4*** **:MCI**	−157.13	0.02
***ApoE2*** **:AD**	−512.69	0.20
***ApoE2*** **:MCI**	35.55	0.79

n = 662.

Results for model which treats APOE epsilon “X” allele effects as dose-dependent effects. APOE effect sizes of each variable are reported in terms of mm^3^/(unit variable), e.g., the effect of “Age” on the outcome variable, hippocampal volume, is −23.31 mm^3^/year of age. “:” denotes interaction terms between variables. Reference for the model is an APOE ε3/ε3 CN individual. Note the lack of a main effect of APOE epsilon 4 (“ApoE4”) in the model and thus in CN subjects.

### Hippocampal Volume Model–APOE ε4 Effects

**Figure 2:**
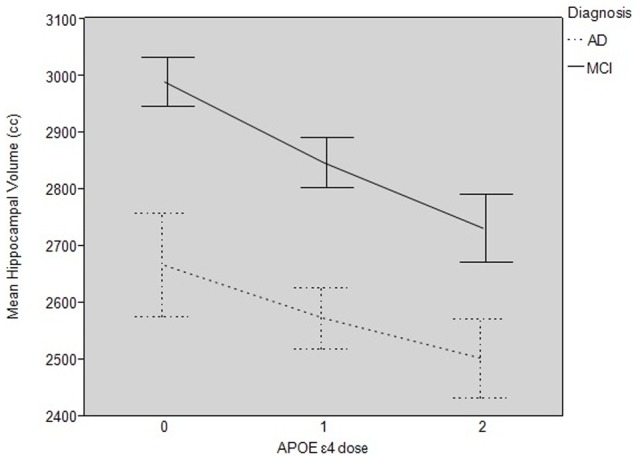
Dose-response of hippocampal volumes. Dose-dependency of hippocampal volume by *APOE* epsilon 4 allele load in Mild Cognitive Impairment (MCI) and Alzheimer Disease (AD). Each error bar is constructed using ±1 standard error of the mean.

When modeling the effects of the *APOE* genotype as a continuous variable, we found that there was no main effect of the *APOE* ε4 allele on hippocampal volume, and thus no dose-dependent effect of ε4 in CN controls (because the reference subject in the model is an *APOE* ε3/ε3 CN subject, the main effects listed in the model output are those used to explore effects in CN individuals, e.g. “Age” in Table 7 denotes the effect of age on hippocampal volume in an *APOE* ε3/ε3 CN subject). However, there were significant dose-dependent interactions between ε4 and diagnoses of AD and MCI; specifically, the model revealed that the interaction of a diagnosis of AD and ε4 dose resulted in hippocampal volumes that were 152.7 mm^3^ smaller (−4.0%, Cohen’s *d* = −0.16, effect size coefficient *r* = 0.75) per ε4 allele in AD as compared to AD individuals with no ε4 alleles (*p* = 0.04). There was a similar dose-dependent interaction between a diagnosis of MCI and ε4 dose, such that hippocampal volumes were atrophied by an additional 157.13 mm^3^ (−4.0%, Cohen’s *d* = −0.19, effect size *r* = 0.67) per ε4 allele in MCI as compared to MCI subjects with no ε4 alleles (*p* = 0.02). Figure 2 depicts the dose-response relationship between ε4 allele load and hippocampal volume as seen in MCI and AD subjects.

### Hippocampal Volumes–APOE ε2 Effects

When modeling the effects of the *APOE* genotype as a continuous variable, we found that there was a significant, dose-dependent, main effect of the *APOE* ε2 allele. In this dose-dependent relationship, each allele accounts for a 769.3 mm^3^ (+19.8%, Cohen’s *d* = 0.23, effect size *r* = 0.62) increase in hippocampal volume (*p*<0.0001) as compared to the model-predicted reference mean for CN controls with no ε2 alleles. See Table 7 for a summary of the results of the Hippocampal Volume Model 1.1.

## Discussion

The current study reveals an effect of the *APOE* ε4 allele for greater hippocampal atrophy in subjects with a diagnosis of MCI or AD as compared to individuals without the ε4 allele in these diagnostic cohorts. That is to say, subjects with the *APOE* ε4 genotype have significantly worse atrophic changes seen in the hippocampi as Alzheimer-spectrum pathology progresses. Furthermore, not only does presence of ε4 correlate with greater atrophy, but the extent to which it is greater is dose-dependent with respect to the number of copies of ε4 that are present, i.e. it is linearly proportional to allele load. Notably, we were unable to demonstrate this effect in CN controls. It is difficult to say why this is the case given several previous reports of *APOE* ε4-driven effects in the hippocampus of cognitively normal controls (see [12] for example). It is possible that this is due to insufficient statistical power; in fact, there were only *n* = 5 CN ε4 homozygotes that met inclusion criteria for the study. This small number of homozygotes, in conjunction with the fact that our model was constrained to the assumption of dose-dependency with regard to allele load, could potentially explain why the results from the dose-dependent model (Table 7) do not detect an overt *APOE* ε4 effect. This result is consistent with findings of several recent studies including those of Crivello *et al* [41] who showed that there is no *APOE* ε4 dose-effect on longitudinal hippocampal atrophy rates in healthy controls, and Tupler *et al* whose study of 159 CN elderly subjects revealed no difference in baseline hippocampal volume according to *APOE* ε4 status [42]. However, given the discordance between our findings and those of several previous reports, it is ultimately unclear as to the reason an *APOE* ε4-effect was not seen in CN individuals.

While effects of ε4 on the hippocampus have been found previously [10]–[14], the demonstration of a dose-response is new to our knowledge, and differs from three previous studies that have examined this issue: Filippini *et al*. showed that the *APOE* ε4 allele exerts a dominant rather than dose-dependent effect on decreased deep gray-matter volumes, and neither Lemaitre H. *et al* nor Liu Y. *et al.* found a specific dose-dependent relationship as we have defined it here [43]–[45]. Issues related to sample size, variations in population ε4 allelic frequencies and demographic characteristics, non-uniformity across volumetric-derivation methods, and lack of fully-automated algorithms are possible sources of variation that could account for these discrepancies.

Our conclusions regarding ε2-driven effects are not as certain. For instance, though our model indicates a significant dose-dependent main effect of the ε2 allele for moderate hippocampal protection, this result seems to be driven almost exclusively by the only two ε2 homozygotes in the study, both CN subjects with exceptionally large hippocampi. Though statistically valid according to our analysis, it is difficult to generalize from such a small number of subjects. It is worth noting, however, that using a slightly altered model–which treats *APOE* as a nominal or categorical (*carrier*/*non*-*carrier*) variable–in order to re-analyze a sub-set of this data which excludes these two ε2 subjects: 1) does not significantly alter the ε4 effects seen, and 2) still generates results suggestive of a protective morphometric main effect of a single ε2 allele (an effect size of +505.84 mm^3^, equivalent to a nearly 13% increase in hippocampal volume; Cohen’s *d* = 0.14, effect size *r* = 0.68)–though, this result is only marginally significant (*p* = 0.07) (see Table 8 for a full summary of the results for this modified model). Thus, though our results are highly suggestive and may serve to guide future inquiry into the question, it currently may be difficult to draw robust conclusions regarding the putative protective effect the ε2 allele has on hippocampal morphometry. These results differ somewhat from previous studies examining ε2 effects, none of which were able to find a significant difference in hippocampal volumes according to *APOE* ε2. This includes both those that relied on manual-only volumetry [12], [13] as well as even more recent studies using fully-automated methods [21], [22]–the latter two both having reported non-significant trends for larger mean hippocampal volumes for ε2 carriers versus non-carriers.

**Table 8:** Summary of effects for variables on hippocampal volume: *APOE* nominal model.

	Effect (mm^3^)	*p*-value
**(Intercept)**	3914.39	<0.0001
**Right side**	41.26	<0.0001
**ApoE41**	−35.23	0.6
**ApoE42**	75.32	0.68
**ApoE21**	505.84	0.07
**ApoE22**	*n/a*	*n/a*
**Age**	−23.61	<0.0001
**AD**	−583.93	<0.0001
**MCI**	−360.92	<0.0001
**ICV**	183.09	<0.0001
**ApoE41:AD**	−96.14	0.35
**ApoE42:AD**	−392.67	0.05
**ApoE41:MCI**	−131.14	0.12
**ApoE42:MCI**	−392.61	0.05
**ApoE21:AD**	−414.15	0.08
**ApoE21:MCI**	113.42	0.43
**ApoE21:Age**	−18.17	0.08

n = 660; n = 2 APOE ε2 CN homozygotes excluded. Results for model which treats APOE epsilon “X” allele effects as categorical or “carrier-status” effect. APOE effect sizes of each variable are reported in terms of mm^3^/(unit variable). “APOEX1” denotes the effect of 1 copy of APOEX, “APOEX2” denotes the effect of 2 copies of APOEX. Unlike the results of the above model, this model does not have the assumption of dose-dependency.

“:” denotes interaction terms between variables. Reference for the model is an APOE ε3/ε3 CN individual.

There are limitations to the current study. Firstly, the number of ε4 (*n* = 5) and ε2 (*n* = 2) homozygotes included in our analyses was less than ideal; as mentioned, the presence of only two ε2 homozygotes makes it difficult to generalize from any results regarding an ε2 dose-effect, and similarly, ε4-driven effects in CN subjects may have been below our threshold for detection given the low number of ε4 homozygotes in conjunction with the constraints imposed by the dose-dependent model. Secondly, the overall ε2 allele frequency in the present study (48/1324; 3.6%) was low for one which set out to test for a main effect of this allele. Thirdly, though the ADNI study allows for examination of large study cohorts, this particular group of subjects has previously been shown to have a higher proportion of whites, to be freer of co-morbid conditions, and to be more educated than community-based samples [24], which complicates generalizations of findings using ADNI data. Finally, there are the limitations imposed by the cross-sectional study design, namely, the limitation that there can be no inferences made with regard to causality. Specifically, we have used the term “atrophy” throughout our paper to refer to ε4-driven effects for smaller hippocampal volumes; although our data are consistent with other longitudinal data for atrophy rates, our cross-sectional design is unable to determine if such volume differences are due to accelerated atrophy, or simply a genetic trait marker.

Despite these limitations, the results of this study have implications for our understanding of the mechanisms underlying the well-documented *APOE* ε4-mediated acceleration of AD pathophysiology. Specifically, the dose-effects shown here are consistent with the mechanisms described by Jiang *et al.,* among others, in which the ε4 isoform of ApoE results in a protein markedly less able to fulfill its neuro-protective tasks–tasks that are much more readily accomplished by the ε2 and ε3 isoforms–such as the proteolytic degradation of extracellular amyloid-β in the brain [7].

Future directions should include re-examination of this issue with a more substantial and statistically robust cohort that includes a higher number of individuals possessing the alleles of interest. Also of further importance would be to explore this cohort for any dose-dependent effects of the ε4 and ε2 alleles on the longitudinal atrophy rates of these measures, with the goal of using *APOE* genotype to better interpret quantitative, longitudinal imaging data for prognostic purposes.
